# Antimicrobial Activity and Resistance: Influencing Factors

**DOI:** 10.3389/fphar.2017.00364

**Published:** 2017-06-13

**Authors:** Jun Li, Shuyu Xie, Saeed Ahmed, Funan Wang, Yufeng Gu, Chaonan Zhang, Ximan Chai, Yalan Wu, Jinxia Cai, Guyue Cheng

**Affiliations:** ^1^MOA Laboratory for Risk Assessment of Quality and Safety of Livestock and Poultry Products, Huazhong Agricultural UniversityWuhan, China; ^2^National Reference Laboratory of Veterinary Drug Residues (HZAU) and MOA Key Laboratory for The Detection of Veterinary Drug Residues in Foods, Huazhong Agricultural UniversityWuhan, China; ^3^Basic Veterinary Medicine, College of Veterinary Medicine, Huazhong Agricultural UniversityWuhan, China

**Keywords:** resistance, persistence, antibiotic concentration, inoculum size effect, serum effect, gut microbiota

## Abstract

Rational use of antibiotic is the key approach to improve the antibiotic performance and tackling of the antimicrobial resistance. The efficacy of antimicrobials are influenced by many factors: (1) bacterial status (susceptibility and resistance, tolerance, persistence, biofilm) and inoculum size; (2) antimicrobial concentrations [mutant selection window (MSW) and sub-inhibitory concentration]; (3) host factors (serum effect and impact on gut micro-biota). Additional understandings regarding the linkage between antimicrobial usages, bacterial status and host response offers us new insights and encourage the struggle for the designing of antimicrobial treatment regimens that reaching better clinical outcome and minimizing the emergence of resistance at the same time.

## Introduction

Currently, antibiotics are the invaluable weapons to fight against infectious diseases. However, the outbreak of antimicrobial resistance, jointly with the shortage of newly developed antimicrobial drugs, brings a great threat to the health of both human and animals (Cheng et al., 2016). Rational uses of antibiotics are the key approaches in tackling of the antimicrobial resistance. The effectiveness of antibiotic treatment is determined by many factors, mainly from three aspects, the antibiotic itself, the target pathogen, and the patient body system. In this review, factors influencing the antimicrobial activity have been discussed. These factors include consideration of bacterial status, inoculum size, antibiotic concentrations, serum effect, and interaction with the host gut microbiota. Host dispositions of antibiotics including the metabolism, transport processes, and diffusion between different compartments have been fully discussed in several reviews (Czock et al., 2009; Jarrell et al., 2015), therefore they are not discussed here except the antibiotic-protein binding which leads to the serum effect. These insights could be useful to design more effective clinical antibiotic therapy (Estes, 1998).

## Bacterial status

Bacterial status is one of the determinants for antimicrobial activity. The bacterial phenotypes are different under antibiotic exposure, such as susceptibility, resistance, tolerance, and persistence (Brauner et al., 2016).

### Susceptibility and resistance

Susceptibility and resistance is measured by the minimum inhibitory concentration (MIC). MIC is defined as, the minimum concentration of an antibiotic to inhibit the bacterial growth. MIC is usually determined by exposing a defined amount of bacterial population to a series of increasing antibiotic concentrations in a standardized growth medium for about 16–20 h (Wiegand et al., 2008). Isolates can be phenotypically recognized as susceptible and resistant according to the epidemiological cut-off (ECOFF) value or breakpoint (Cheng et al., 2016).

Clinical resistance is a condition in which the clinical criteria of cure did not reached, when a sufficient antibiotic dosage and administration timetable are applied for a specific infection. Clinical resistance is determined by the clinical breakpoints, which separates clinically resistant (related with a high possibility of therapeutic malfunction) from a clinically susceptible bacteria (related with a high possibility of therapeutic victory; Turnidge and Paterson, 2007). Clinical breakpoints are influenced by pharmacokinetic/pharmacodynamic (PK/PD) parameters, which indicate the relationship between antimicrobial activity *in vivo* and the antibiotic concentration at the site of infection. The PK/PD breakpoint which includes methods of animal models and statistical techniques which indicates a target achievement with a high probability of treatment outcome (Turnidge and Paterson, 2007). Clinical breakpoints are usually defined regarding to the criteria which is established by the Clinical and Laboratory Standards Institute (2011) and the European Committee on Antimicrobial Susceptibility Testing (2011).

### Tolerance

**Figure 1:**
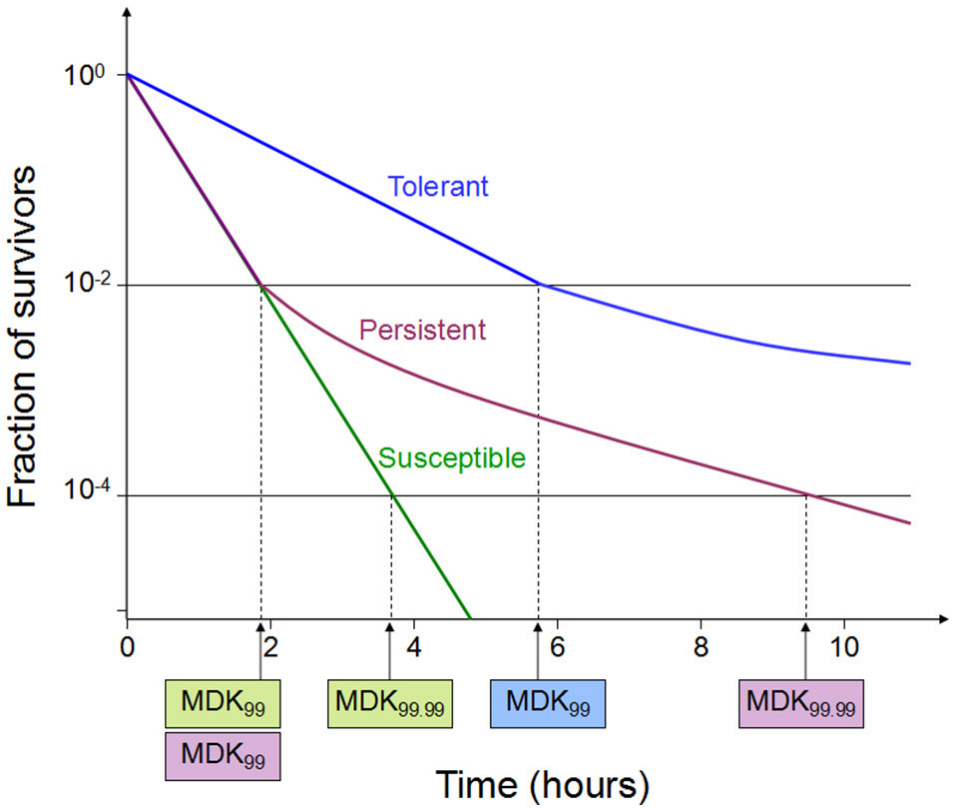
Time-kill curves of susceptibility, tolerance and persistence (modified from Brauner et al., 2016). A persistent strain of bacteria has a similar MIC and a similar MDK99 to a susceptible strain; however, the MDK for 99.99% of bacterial cells in the population (MDK99.99) is substantially higher for a persistent strain than the MDK99.99 for a susceptible strain. The minimum duration for killing [MDK; for example for 99% of bacterial cells in the population (MDK99)] for a tolerant strain is substantially higher than the MDK99 for a susceptible strain.

Tolerance is the capacity of a bacteria to stay alive in a fleeting exposure to antibiotics, which applies only to bactericidal antibiotics (Kester and Fortune, 2014). Longer time rather than high concentration of an antibiotic exposure is necessary to construct the same level of killing in a tolerant strain as in a susceptible strain. Tolerant and non-tolerant bacteria may not be different in MIC-value. The minimum duration of killing (MDK) which can be obtained from the time-kill curves are suggested as a quantitative measure of tolerance (Brauner et al., 2016; Table 1, Figure 1). MDK is defined as, the time of an antibiotic treatment essential to kill a known fraction of the bacterial population at an antibiotic concentration that go over the MIC. Likewise to the MIC, that can be used to evaluate the level of resistance between bacterial strains; the MDK can be used to compare the level of tolerance between strains. An evaluation framework that measures both the MDK and the MIC would enable a clear distinction to be made between resistance (an increase in the MIC) and tolerance (an increase in the MDK; Fridman et al., 2014).

**Table 1:** Difference between resistance, tolerance and persistence.

	**Resistance**	**Tolerance**	**Persistence**
Heterogeneity	No	No	Yes
Inheritability	Yes	Yes or No	No
MIC	MIC_R_ > MIC_S_	MIC_T_ = MIC_S_	MIC_P_ = MIC_S_
MDK	–	MDK_99T_> MDK_99S_	MDK_99P_ = MDK_99S_ MDK_99.99P_ > MDK_99.99S_
Form	–	1) Tolerance by slow growth (High MDK in stationary and exponential inocula) 2) Tolerance by lag (High MDK only in stationary inocula)	1) Time-dependent persisters a) Persistence by slow growth b) Persistence by lag
			2) Dose-dependent persisters

*MIC, the minimum inhibitory concentration; MDK, the minimum duration for killing; MDK_99_, MDK for 99% of bacterial cells in the population; MDK_99.99_, MDK for 99.99% of bacterial cells in the population; _S_, Susceptible; _R_, Resistant; _T_, Tolerant; _P_, Persistent*.

There are generally two types of tolerance, “tolerance by slow growth” and “tolerance by lag,” the former occurs at stationary phase while the latter occurs in a transient growth arrest often induced by starvation or stress (Brauner et al., 2016). It has long been known that decreased rate of growth increases the bacterial tolerance to some antibiotics, such as β -lactams and fluoroquinolones. The actions of these drugs require for bacterial growth. “Tolerance by slow growth” may be inherited in some bacterial species, such as *Mycobacterium tuberculosis* (Manina et al., 2015), or non-inherited when the growth of bacteria is damaged by a specific poor conditions (e.g., location in biofilm, exposure to inhibitors; Bernier et al., 2013) and the host factors (Helaine et al., 2010).

The lag phase is the time that growth-arrested bacteria restart the exponential growth when expose to a growth-permissive environment (e.g., bacteria enter into the host environment or control between different niches; Amato et al., 2013). In lag phase the bacterial cells initially adapted the new circumstance before resuming the exponential growth (Madar et al., 2013). Tolerance by lag phase occurs when the duration of the growth arrest is longer than the antibiotic treatment time (Balaban et al., 2004). Tolerance by lag phase can reach an MDK of many hours or days (Fridman et al., 2014). Inherited tolerance by lag phase includes many mutations and a number of tolerome-related genes which are more than resistome-related genes (Girgis et al., 2012), suggesting that the development of high tolerance may take place faster than the evolution of high resistance.

### Persistence

Persistence occurs in a bacterial subpopulation (classically <1%) that are not killed by antibiotics, and heterogeneous response is repeated when they are expose to the same antibiotic (Lewis, 2007). The detection of persisters requires different percentile for the MDK measurement, usually using the MDK_99.99_ (time of treatment duration required to kill 99.99% of a bacterial population; Brauner et al., 2016; Table 1, Figure 1). Dormant persisters are found to be present in a mouse models infected by *S. typhimurium* (Helaine et al., 2010) or *M. tuberculosis* (Manina et al., 2015). Time-dependent persistence is defined as the bacteria which typically has either a longer lag time or slower growth rate than the majority of the population (Balaban et al., 2004). The molecular mechanisms of time-dependent persistence are also associated with tolerance that slows down the killing by antibiotics (Adams et al., 2011).

MDK provide an apparent predictions of the treatment period that is needed to treat an infection by strains with the tolerance or time-dependent persistence, and could thus be combined with PK/PD models to optimize treatment regimens (Brauner et al., 2016). However, in some case of the tolerance with very high MDK, the antibiotic toxicity to host may limit the treatment duration. Dose-dependent persistence might be treated with inhibitors, such as efflux pump inhibitors (Adams et al., 2014).

Drug-induced tolerance or persistence which causes growth-arrest in some of the microorganisms may results in a long MDK (Dorr et al., 2010; Johnson and Levin, 2013). Survival of the bacterial population under antibiotics may facilitate the subsequent emergence of resistance, e.g., increase the mutation rates. Understanding the bacterial survival strategies gives a better understanding of how bacteria evolve resilience to antimicrobials (Cohen et al., 2013).

### Biofilm

Microorganisms can grow up in a free-living (planktonic) or in a cell aggregates (biofilm). Biofilms are consortium of bacteria, which are surrounded in a self producing polymer matrix which consist of a polysaccharides, proteins and DNA (Hall-Stoodley et al., 2012). Host factors such as platelets, immunoglobulin, and fibrin, may also be included into the extracellular matrix (Akiyama et al., 1997). Antimicrobial resistance can emerge in a biofilms by at least three different mechanisms: (1) impair the antibiotics diffusion of into the surrounded bacterial cells by extracellular matrix; (2) lesions in the mismatch repair system or in the DNA oxidative repair system resulting in hypermutator; and (3) emergence of persistent bacterial cells (Penesyan et al., 2015). High cell density in a biofilms may increase the number of resistant mutants that can be selected under antibiotic pressure and the extracellular DNA in biofilm matrix can facilitate horizontal gene transfer of resistance determinants (Cheng et al., 2016). Based on the results of *in vitro* studies of Yonezawa et al. (2015), *Helicobacter pylori* biofilm formation can reduce sensitivity to antibiotics and resistance mutations are more often generated in a biofilms than in a planktonic cells. Zhang et al. examined the correlation between biofilm and antibiotic resistance among 110 strains of clinical isolates of *Haemophilus parasuis*. The results indicated that *H. parasuis* field isolates have the capability to form biofilms *in vitro*. In addition, biofilm positive strains have a positive association with a resistance against β -lactams antibiotics and may play an important role in *H. parasuis* infections (Zhang et al., 2014). Using an *Escherichia coli* biofilm model, Tyerman et al. demonstrated that a heritable variation for the broad-spectrum antibiotic resistance can arise and accumulate rapidly during biofilm development, even in the absence of antibiotic selection (Tyerman et al., 2013). A study by Bae et al. reported that *Campylobacter jejuni* transfers antibiotic resistance genes more frequently in biofilms than in planktonic cells by natural transformation (Bae et al., 2014).

Molecular and structural understanding of biofilm has led to the advances in targeting the specific biofilm determinant mechanism, such as anti-adhesion, targeting signaling pathways, dispersing biofilm matrix, and eliminating persisters, which could be applied in a combination with the antibiotic therapy (Cheng et al., 2014, 2016).

## Bacterial inoculum size

Inoculum effect is defined as an increase in the MIC with increasing bacterial inoculum size (Brook, 1989). If there is an inoculum effect, bacteria might appear as a susceptible when the inoculums is standard (10^5^ CFU/ml) but resistant if the inoculum size is increased. Several studies have been shown that high bacterial inocula at the infection sites may decrease the activity of antibiotic (Table 2). The mechanisms underlying the inoculum effect may be reduced ratio of available drug molecules per target because of reduced effective antimicrobial concentration (Udekwu et al., 2009). Inoculum size is also important in the emergence of an antibacterial resistance. At marbofloxacin concentrations within the MSW, the appearance of *E. coli* mutants resistant to marbofloxacin was more frequent when the initial size of the bacterial population was increased, indicating that the process of a mutant selection within the MSW was influenced by the presence of mutants before any antibiotic treatment (Ferran et al., 2007).

**Table 2:** Recent studies on inoculum size effect of antibiotics.

**Bacteria**	**Inoculum size**	**MIC Change**	**References**
*E. coli* ATCC 25922 (non-ESBL producer) *E. coli* Ec1062 (CTX-M-14 producer)	10^5.5^ CFU/g and 10^7.5^ CFU/g	In an experimental murine sepsis model, piperacillin-tazobactam and imipenem reduced spleen ATCC 25922 strain concentrations (−2.53 and −2.14 log10 CFU/g [*P* <0.05, respectively]) in the HI vs. LI groups, while amoxicillin-clavulanate maintained its efficacy (−1.01 log10 CFU/g [no statistically significant difference]). Regarding the Ec1062 strain, the antimicrobials showed lower efficacy in the HI than in the LI groups: −0.73, −1.89, and −1.62 log10 CFU/g (*P* <0.05, for piperacillin-tazobactam, imipenem, and amoxicillin-clavulanate, respectively, although imipenem and amoxicillin-clavulanate were more efficacious than piperacillin-tazobactam).	Docobo-Perez et al., 2013
*E. coli*	5 × 10^2^ to 5 × 10^8^ CFU/ml	The concentrations of marbofloxacin needed to eradicate all bacterial population increased from 1- or 2-fold the MIC for low inocula to 128- or 256-fold the MIC for the 5 × 10^7^ and 5 × 10^8^ CFU/ml inocula.	Ferran et al., 2014
MSSA & MRSA	2.5–4 × 10^2^ to 2.5–4 × 10^6^ CFU/spot	To MSSA, a big IE for ampicillin; small IE for cefazolin, meropenem, and ciprofloxacin; middle IE for teicoplanin and linezolid.	Miyake et al., 2011
		To MRSA, small change in vancomycin and arbekacin; middle change in teicoplanin and linezolid.	
MRSA	10^4^, 10^6^, and 10^8^ CFU/ml	A small IE for vancomycin (MIC_L_ = 1 mg/ml, MIC_M_ = 1–2 mg/ml, and MIC_H_ = 2 mg/ml); a significant IE for daptomycin (MIC_L_ = 0.25 mg/ml, MIC_M_ = 0.25–0.5 mg/ml, and MIC_H_ = 2 mg/ml); no IE for linezolid at low and medium inocula (MIC_L_ = 1 mg/ml and MIC_M_ = 1–2 mg/ml), but with the high inoculum, concentrations up to 2,048 mg/ml did not fully inhibit visual growth.	Rio-Marques et al., 2014
*Staphylococcus aureus Pseudomonas aeruginosa*	10^5^–10^8^ cfu/mL	10^5^~10^8^ cfu/mL had no significant effect on the MICs of fluoroquinolones and carbapenems; however, inoculum size to >10^8^ cfu/mL resulted in a reduction in bactericidal activity against *S. aureus*; increasing the inoculum size of *P. aeruginosa* exerted only a minimal influence on the bactericidal activity of fluoroquinolones, but resulted in a reduction in the bactericidal activity of carbapenems; when the inoculum was increased above 10^6^ cfu/mL, the duration of the PAEs of these antimicrobial agents was reduced; Inoculum size had a greater influence on the *in vivo* efficacy of carbapenems than that of fluoroquinolones in mouse model.	Mizunaga et al., 2005
*P. aeruginosa*	10^6^, 10^8^, and 10^9^ CFU/ml	The killing of the susceptible population was 23-fold slower at the 10^9^ CFU/ml and 6-fold slower at the 10^8^ CFU/ml than at the 10^6^ CFU/ml.	Bulitta et al., 2010
*Staphylococus* spp *Streptococcus* spp. *Enterobacteriaceae P. aeruginosa*	5 × 10^3^, 5 × 10^5^, and 5 × 10^7^ CFU/ml	An increase over 7-fold of the MIC in ozenoxacin, ciprofloxacin, and levofloxaci at 10^7^ CFU/mL	Tato et al., 2014
*Pasteurellaceae*	5 × 10^5^ and 5 × 10^8^ CFU/ml	Marbofloxacin was equally potent against 10^5^ CFU/mL inocula *Mannheimia haemolytica* and *Pasteurella multocida*; an IE was observed with *P. multocida* at a 10^8^ CFU/mL inoculum; no IE was observed with *M. haemolytica*. At the same dose, the clinical and bacteriological outcomes were much better for mice infected with *M. haemolytica* than for those infected with *P. multocida* with 10^9^ CFU of each bacteria	Lhermie et al., 2015
*Klebsiella pneumoniae*	10^5^ CFU or 10^9^ CFU/animal	The dose of 50 mg/kg b.w. cefquinome targeting the high *K. pneumoniae* inoculum cured all the treated rats and resulted in a massive amplification of CTX-M-producing *Enterobacteriaceae*. A dose of 5 mg/kg targeting the low *K. pneumoniae* inoculum cured all the rats and averted an outbreak of clinical disease, all without any amplification of CTX-M-producing Enterobacteriaceae.	Vasseur et al., 2014

*ESBL, extended-spectrum β-lactamase; MSSA, methicillin-susceptible Staphylococcus aureus; MRSA, methicillin-resistant Staphylococcus aureus; IE, inoculum effect*.

Another reason for inoculum effect regarding the reducing of antibiotic potency is due to the self-limiting of a bacterial growth in the high inoculum size which may increase bacterial tolerance to some antibiotics or the presence of heterogeneity resistant persisters in such inocula. When 5 × 10^3^ CFU/ml of *gyrA* mutant *E. coli* were mixed with 5 × 10^7^ CFU/ml of wild-type bacteria, MPC could eradicate both of the bacteria; whereas when 5 × 10^3^ CFU/ml of *gyrA* mutant *E. coli* were mixed with a 1-log_10_ higher inoculum (5 × 10^8^ CFU/ml) of wild-type bacteria, the mutants were not eliminated when expose to antibiotic concentrations above the MPC (Ferran et al., 2014). Lee et al. (2010) suggests that a small number of bacterial resistant mutants can provide the protection to others by producing signaling molecule indol to turn on the drug efflux pumps and oxidative-stress protective mechanisms, enhancing the survival capacity of the overall population.

Inoculum size could have considerable affect on the pharmacokinetic/pharmacodynamic parameters of antibiotics. In a mouse thigh model challenged with either a high (10^8^ CFU) or a low (10^5^ CFU) inoculum of *E. coli*, the values of time within the mutant selection window (*T*_MSW_) <30% appeared to be a good predictor for prevention of a resistance (Ferran et al., 2009). When the time within the MSW was higher than 34%, the selection of resistant bacteria occurred more often in thighs initially infected with the high inoculum (80%) than in those infected with the low inoculum (46%; Ferran et al., 2009). In another study, a rat lung infection model was challenged with a low (10^5^ CFU) or a high (10^9^ CFU) inoculum of *Klebsiella pneumonia*, the results displayed that for the low inoculum, prevention of resistance occurred for an AUC/MIC ratio of 189 h, while for the high inoculums, resistant bacteria were enriched for AUC/MIC ratios up to 756 h (Kesteman et al., 2009). For rats infected with a high inoculums size, the parameters of AUC/MIC, *C*_max_/MIC, and *T*_MSW_ were not found to be effective predictors for the resistance prevention. They proposed an original index, the *T*_>MPC_/*T*_MSW_ ratio, which reflects the ratio of the time that the less susceptible bacterial subpopulation is killed over the time that it is selected and this ratio is valid only if the plasma concentrations achieve the MPC (Kesteman et al., 2009).

Different antibiotics show different inoculum effects on the growth and selection of resistance of the same strain. A small inoculum effect was observed for vancomycin and a significant inoculum effect for daptomycin in methicillin-resistant *Staphylococcus aureus* (MRSA), while linezolid exhibited no inoculum effect at low and medium inocula (10^4^ and 10^6^ CFU/ml) but with the high inoculum (10^8^ CFU/ml; (Rio-Marques et al., 2014)). After incubation with either drug at 2-fold increasing concentrations for 15 days, MICs of low, medium, and high inocula to vancomycin were 2–4, 4–8, and 4–16 mg/L and for daptomycin were 0.5~2, 8~128, and 64~256 mg/L, respectively (Rio-Marques et al., 2014). In addition, one antibiotic may show differential inoculum effect against different strains. It was demonstrated that marbofloxacin was equally potent against 10^5^ CFU/ml inocula of *Mannheimia haemolytica* and *Pasteurella multocida*. However, an inoculum effect was observed with *P. multocida*, whereas there was no inoculum effect for *M. haemolytica* (Lhermie et al., 2015). The *in vivo* mice model infected with 10^9^ CFU of each bacteria also showed that the clinical outcomes of marbofloxacin were much better for mice challenged with *M. haemolytica* than those infected with *P. multocida* (Lhermie et al., 2015).

In order to summarize, for the bacterial populations of high inoculum, both wild-type and resistant bacteria are with very low rates of division, therefore the antimicrobial activity is dramatically reduced and targeting the mutant bacteria to improve the clinical outcomes in a patients is not enough (Ferran et al., 2014). In a clinical settings, there is a need for a prompt antibiotic treatment to minimize the inoculum size. Meanwhile, strategies aimed at lowering the inoculum size at the infection site should be used whenever possible in parallel to antimicrobial therapy (Rio-Marques et al., 2014). This highlights the importance of surgical drainage or infection source removal in high bacterial density infections (Entenza et al., 2010). Moreover, bacterial species-specific antibiotic dosing schedules is needed in a clinical settings (Lhermie et al., 2015).

## Antibiotic concentrations

The antibiotics apply its effect by different mechanisms, initially by inhibiting the synthesis of the bacterial wall (penicillins, glycopeptides, carbapenems, and cephalosporins), inhibiting DNA replication (quinolones) or its transcription (rifampicin), impairing bacterial ribosomes and protein synthesis (macrolides, linezolid, dalfopristin, tetracyclines, and aminoglycosides), interfering with metabolic pathways (sulfonamides and trimethoprim) or disrupting the cytoplasmic membrane (polymyxin and daptomycin; Zamoner et al., 2016). Different antibiotic concentrations may results in a different selection of the resistant bacteria, thus influencing the efficacy of antimicrobials.

### Mutant selection window (MSW)

The antimicrobial choices enrich the resistance genes which are already present earlier than selection operates in a particular setting. Susceptible population will be inhibited at an antibiotic concentration above the MIC. Resistant isolates should be inhibited by a higher concentration (i.e., the MICs of the resistant mutants). Mutant selection window (MSW) is a collection of concentrations between the MICs of the susceptible and resistant variants. Resistant mutants may be selected under antimicrobial selective pressures in the MSW (Drlica, 2003). The upper boundary, defined as the mutant prevention concentration (MPC), can inhibit the growth of the entire bacteria population (Drlica and Zhao, 2007). Determination of MPC needs a large inoculum (approximately 10^9^~10^10^ CFU/ml) compared to MIC testing (10^5^ CFU/ml; Blondeau, 2009). This high inoculum is applied to ensure the emergence of the first-step mutants. The MPC concept can also be applied to higher-order mutants.

The MSW concept aims to the prevention of a resistance. When the pertinent antimicrobial agent is present at a concentration within the MSW, a selection process of resistance will occur. Therefore, dosages ensuring antibiotic concentrations at the infection site above the MPC are suggested. The emergence of resistant bacteria is a dynamic phenomenon over time, the MPC should be established with new PK/PD knowledge (Mouton et al., 2011). And also high doses may impose a potential toxicity to human or animals thus its limits the clinical usage of the MPC concept.

### Sub-inhibitory concentrations

Antibiotic at sub-inhibitory concentrations are found in many of the natural environments, such as soil and water, they are also generated as a consequence of antibiotic therapy in a humans and livestock, such as suboptimal dosing therapy, poor pharmacokinetics, usage of low-quality drugs, and a poor patient compliance (Andersson and Hughes, 2014). In agricultural sector, antibiotics are often administered as a feed additives to promote growth of animals, the doses are typically sub-therapeutic and often result in a concentrations below the MIC (Wallinga and Burch, 2013), which is termed as sub-MIC concentrations. This concentration allows susceptible strains to continue growing at a reduced growth rate.

**Figure 2:**
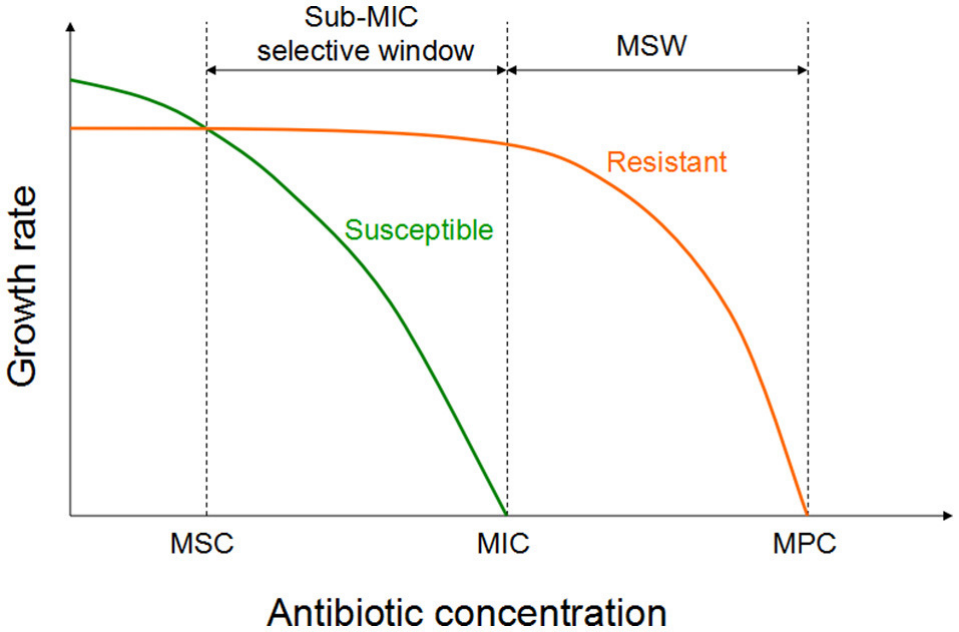
Growth of the susceptible and the isogenic resistant bacteria in the sub-MIC selective window and traditional MSW (modified from Gullberg et al., 2011). For antibiotic concentrations lower than MSC, the susceptible strain (green line) will outcompete the resistant strain (orange line). However, for the antibiotic concentrations between sub-MIC selective window and traditional mutant selective window, the resistant strain will outcompete the susceptible strain.

Recent investigation have been shown that sub-MIC concentrations of antibiotic can be choose for a low-level of resistance, which eventually serve as a stepping stones paving the way for high-level resistance (Baquero et al., 1998; Baquero, 2001). Two recent studies investigated the selective potential of sub-MIC concentrations (Gullberg et al., 2011; Liu et al., 2011). In one of the experiment, researchers mixed wild-type and isogenic resistant bacterial strains with a single resistance mutation or resistance gene at an initial mutant/wild type ratio of 1/1 (Gullberg et al., 2011). As shown in Figure 2, in the antibiotic absence, wild-type strain had a competitive advantage against the resistant strain (due to fitness cost of particular resistance determinant). Though the antimicrobial concentration increased, a progressive shift has been started in the selection toward the isogenic resistant strain. The lowest antimicrobial concentration is needed to choose for the resistant mutant over the wild type is called as the minimal selective concentration (MSC). Selection for the resistant mutants also occurs at a concentrations of the sub-MIC selective window (between the MSC and the MIC of the susceptible strain; Figure 2; Gullberg et al., 2011).

Except for enriching of pre-existing resistant variants, sub-MIC concentration can also select for *de novo* resistance by increasing the rate of adaptive evolution, including a resistance development. Several classes of antibiotics at a sub-MIC levels (such as fluoroquinolones, β -lactams, and aminoglycosides) have been reported to be able to induce the SOS response and the RpoS regulon in bacteria (Gutierrez et al., 2013), which can leads to a genetic alterations including movement of mobile elements that may carry resistance or virulence determinants (Beaber et al., 2004; Ubeda et al., 2005), activation of recombinases (Lopez et al., 2007; Lopez and Blazquez, 2009), and increase in the rate of mutagenesis during chromosome replication (Kohanski et al., 2010; Baharoglu and Mazel, 2011; Thi et al., 2011).

In addition, it has been shown that antibiotic-associated signaling can influence the resistance development in a bacterial population (Lee et al., 2010). Such signaling resulted in a several functional consequences including the induction of conjugative transfer, gene expression, quorum sensing, biofilm formation and bacterial virulence (Yim et al., 2007; Dietrich et al., 2008; Fajardo and Martinez, 2008; Romero et al., 2011). A study showed that under an increasing concentrations of norfloxacin, highly resistant *E. coli* population excreted indole as a signaling molecule to the susceptible population, which cause the susceptible strains to up regulate the efflux pump expression, resulting in a population-wide resistance (Lee et al., 2010). Although, there is no evidence that the antibiotic itself can function as a signal, but it led to the selection of bacteria that can produce signal, which, in turn, increased an MIC of the entire population (Andersson and Hughes, 2014).

## Serum effect

Increase of the MIC due to the antibiotics highly bound to proteins in the serum is a phenomenon that has been investigated not only for some old small molecular antibiotics such as cefonicid, cefoperazone, and ceftriaxone (Chambers et al., 1984; Jones and Barry, 1987; Lam et al., 1988) but also for newly developed antibiotics (Kaplan et al., 2013) as well as for the peptides antibiotics such as daptomycin (Lee et al., 1991) and vancomycin (Dykhuizen et al., 1995). It has been documented that only the non-protein-bound fraction of an antimicrobial is microbiologically active (Beer et al., 2009) and drugs with the higher protein binding typically display higher modifications of *in vitro* activity in the presence of human serum (Zeitlinger et al., 2011).

Different antibiotics in the same class may show different serum effects. MICs of ceftriaxone in the presence of human serum were 4- to 8-fold higher than the those obtained in a broth (Jones and Barry, 1987). while, the bactericidal activity of cefotaxime, desace tylcefotaxime, and cefotaxime plus desacetylcefotaxime were slightly improved in human serum (Jones and Barry, 1987). The activity of ozenoxacin, a new des-fluoro-(6)-quinolone, decreased by increasing the presence of human serum in the medium, but no significant effects with ciprofloxacin and levofloxacin were found (Tato et al., 2014). This is because that most of the fluoroquinolones exhibit low protein binding, ranging from ~20 to 40% in serum (Bergogne-Berezin, 2002), but ozenoxacin exhibited high protein binding, ranging from 85.2 to 86.7% independent of concentration (Tato et al., 2014).

In some of the cases, serum could also display a paradoxical effect on the antibiotic activity. The activity of amphotericin B against *C. albicans* ATCC 24433 was potentiated in RPMI medium in small amounts of the serum, while attenuated at higher concentrations of serum (Richie et al., 2012). This is also the same case for lantibiotic MU1140 against *S. aureus*, with the maximum bactericidal effect at 25% serum concentration (Ghobrial et al., 2010).

The serum effect of the same antibiotic varies between microorganisms. The activity of lantibiotic MU1140 against *S. pneumonia* was inhibited in serum, while it was found to be enhanced against *S. aureus* (Ghobrial et al., 2010). Hypothetically, the augmentation of antimicrobial activity for *S. aureus* could be due to the interaction of serum components with specific extracelluar sites on *S. aureus*, thus acting as a docking site for MU1140 and facilitating the MU1140–lipid II interaction (Ghobrial et al., 2010). The effects of serum on the activity of azoles and amphotericin B reported is also varied, depending upon the fungal strains used (Zhanel et al., 2001).

Although the changes in the antimicrobial activity have been studied at a wide range of serum concentrations (20–100%) in test medium (Zhanel et al., 2001), it should be noted that bacterial growth may be inhibited even at serum concentrations below 50% (Nix et al., 2004). Components such as complement factors transferring and properdin in native serum, might provoke the complex and unpredictable inhibition of bacterial growth (Furbeth and Adam, 1976).

Living systems are a dynamic compared to static *in vitro* tests (Smith et al., 2010), therefore, the results of *in vitro* experiments always require to be assured in *in vivo* settings. For example, telavancin, a glycopeptides, although the protein binding was above 90% and *in vitro* studies observed a 10-fold decline of the activity in the presence of proteins; it was recently permitted on clinical base efficacy (Hegde et al., 2004; Stryjewski et al., 2008). Models should be conducted to mimic physiological states as closely as possible to enable the extrapolate data from a numerous models to *in vivo* state. The establishment of a methods for a direct quantification of affinity of protein binding or its reversibility in individual models may leads to a better understanding of the results where the free drug hypothesis contradict (Zeitlinger et al., 2011).

## Interaction with the gut microbiota

The intestinal microbiota protects the hosts against infections and other pathologies by directly inhibiting colonization of invading microbes or by orchestrating appropriate immune responses, which can control innate and adaptive immunity in the gut (Macpherson et al., 2012; Kabat et al., 2014; Becattini et al., 2016). Commensal bacteria ferment plant-derived fibers producing short-chain fatty acids that feed enterocytes and modulate immune functions, and some of the commensal bacterial species which have the ability to synthesize the essential vitamins that are important for the growth and function of the immune cells (Brestoff and Artis, 2013).

In food producing animals, the most common route of antibiotic administration is oral. However, oral antibiotic administration may results in a gut microbiota dysbiosis, which remains for a long periods of time, spanning months, and even years (Francino, 2015; Gasparrini et al., 2016). Antibiotic treatment during the early postnatal period is one of the most important factors that can influence the maturation of the infant gut microbiota and thus increase the risk of a disease (Cox et al., 2014; Rutten et al., 2015). The usage of antibiotic is found to lead into a reduction in species richness and treatment with specific antibiotics resulted in an enrichment of specific sets of antibiotic resistance genes that are associated with a single species (Gibson et al., 2016; Korpela et al., 2016).

The antibiotics induced microbiota alterations can affect the basic immune homeostasis, especially if they occur early in life, which is an important period for maturation of the immune system and establishment of immunological tolerance (Francino, 2014). The human intestinal microbiota has been recognized as an important reservoir of antimicrobial resistances (Ghosh et al., 2013; Field and Hershberg, 2015; Gasparrini et al., 2016).

Gut microbiota alterations may also increase the sensitivity to infections, which can stem from a lately invaded pathogens or from the abrupt overgrowth and pathogenic actions of the opportunistic organisms which are already exist in the gut (Young and Schmidt, 2004; Sekirov et al., 2010). Besides altering the composition of taxa, antibiotics can also affect the expression of gene, protein activity, and overall metabolism of the gut microbiota (Franzosa et al., 2015). For example, streptomycin generates galactarate and glucarate in the lumen by enhancing the production of host-derived reactive nitrogen species, thereby providing *Enterobacteriaceae* with a fitness advantage (Faber et al., 2016).

On the other hand, some of the antibiotics, except for antimicrobial activity, exhibit immunomodulatory properties. The 14- and 15-member macrolides can interact with mitogen-activated protein kinases, thereby inhibiting NF-κB-mediated inflammatory responses toward various stimuli (Woodhead et al., 2011). Fluoroquinolones exhibiting a cyclopropyl moiety at position N1 of the quinolone core structure may exert anti-inflammatory effects besides their well-established antimicrobial properties (Dalhoff, 2005). Enrofloxacin shows potential effect of on the development of a protective immune response against *H. parasuis* infection (Macedo et al., 2015).

## Conclusions and perspectives

Rational and correct uses of antibiotics are the key approaches in improving antibiotic performance and tackling the antimicrobial resistance. The efficacy of an antibiotic treatment is influenced by many factors. The sensitivity of the specified pathogens is usually combined with pharmacokinetic parameters to investigate the effectiveness of antimicrobial dosage regimens. It should be noted that only the non-protein-bound fraction of an antibiotic is microbiologically active *in vivo*, which makes the serum effect to be considered in an antibiotic therapy. Choosing the precise antibiotic is important, as the serum effect is changed between different antibiotics in the same class or one antibiotic against different microorganisms. Since living systems are more dynamic and complex, the results of *in vitro* tests always necessitate to be assured in *in vivo* settings.

On the other hand, MIC is not informative for some special bacterial status, such as persistent or tolerant bacteria. In contrast to infections caused by planktonic bacteria, biofilm-forming bacteria tend to cause the chronic infections, especially in the respiratory tract, whereby infections persist despite seemingly adequate antibiotic therapy. This is because emergence of persistent or the tolerant bacterial cells is usually happened in biofilms. Recently, several compounds have been identified as an effective against time-dependent persisters (Kim et al., 2011) or against tolerance in biofilms (Fleck et al., 2014) through the method of systematic screens; however, the effectiveness of these compounds has not yet been assessed in the clinical settings. In addition, some existing antibiotics have been found to be less prone to tolerance, such as daptomycin (Mascio et al., 2007).

Antibiotic regimens should be optimized not only for the treatment outcome, but also for the minimization of antimicrobial resistance development (Mouton et al., 2011). When using antibiotics in clinic, there is a requirement for a prompt antimicrobial treatment, which would inhibit the inoculums size enlargement at infection site, as well as for the antibiotic regimens precluding a prolonged period of time within the MSW. Recent studies have also been shown that sub-MIC concentrations can select for resistant mutants. Therefore, we should reduce or prohibit the sub-therapeutic use of antibiotics as the growth promoters or for prophylaxis purposes as much as possible in animal production.

It should not be ignored that antibiotic induced alterations in composition and functions of the microbiota may also create long-lasting harmful effects for the host and increase the bacterial resistance (Francino, 2015; Becattini et al., 2016). Selection of an antibiotic that is less likely to have a long-term effect on the gut microbiota and applying the probiotic bacteria to prevent dysbiosis or to reestablish the gut microbiota after the antibiotic treatment (Browne, 2016; Wischmeyer et al., 2016). In the meantime, we should reduce the unnecessary oral administration of antibiotics to reduce the adverse effects of antimicrobial on gut microbiota. The specified use of bacterial molecules that bind to the specific innate immune receptors is also another strategy to rebuild the interactions altered by antibiotic treatment (Ubeda and Pamer, 2012). For a sustainable antibiotc treatment, the ideal drug should be hydrophilic, of relatively slow clearance, small volume of distribution and have minimal ecological impact on the animal commensal and environmental microbiomes. New, eco-friendly, veterinary AMDs can readily be developed from the currently used drug classes to provide a credible alternative agents (Toutain et al., 2016).

With an improved understanding on the interaction of antimicrobials, pathogens and host gut microbiota and immune response, we have reasons to believe that we will develop judicious antibiotic treatment strategies and better control programs to fight against microbial infections.

## Author contributions

JL and SX wrote and revised the review. SA, FW, YG, CZ, XC, YW, and JC revised the review. GC contributed to the conception of the review and wrote the review.

### Conflict of interest statement

The authors declare that the research was conducted in the absence of any commercial or financial relationships that could be construed as a potential conflict of interest.

## Abbreviations

ECOFF: epidemiological cut-off
HGT: horizontal gene transfer
IE: inoculum effect
MDK: minimum duration for killing
MIC: minimum inhibitory concentration
MSC: minimal selective concentration
MSW: mutant selection window
MPC: mutant prevention concentration
PK/PD: pharmacokinetic/pharmacodynamics.
